# Personalized Mass Production by Hybridization of Additive Manufacturing and Injection Molding

**DOI:** 10.3390/polym13020309

**Published:** 2021-01-19

**Authors:** Praveen Kannan Rajamani, Tatyana Ageyeva, József Gábor Kovács

**Affiliations:** 1 Department of Polymer Engineering, Faculty of Mechanical Engineering, Budapest University of Technology and Economics, Műegyetem rkp. 3., H-1111 Budapest, Hungary; rajamani@pt.bme.hu (P.K.R.); ageyevat@pt.bme.hu (T.A.); 2 MTA-BME Lendület Lightweight Polymer Composites Research Group, Műegyetem rkp. 3., H-1111 Budapest, Hungary

**Keywords:** overmolding, 3D printing, surface modification, bonding strength, mass customization

## Abstract

The new trend in the composites industry, as dictated by Industry 4.0, is the personalization of mass production to match every customer’s individual needs. Such synergy can be achieved when several traditional manufacturing techniques are combined within the production of a single part. One of the most promising combinations is additive manufacturing (AM) with injection molding. AM offers higher production freedom in comparison with traditional techniques. As a result, even very sophisticated geometries can be manufactured by AM at a reasonable price. The bottleneck of AM is the production rate, which is several orders of magnitude slower than that of traditional plastic mass production technologies. On the other hand, injection molding is a manufacturing technique for high-volume production with little possibility of customization. The customization of injection-molded parts is usually very expensive and time-consuming. In this research, we offered a solution for the individualization of mass production, which includes 3D printing a baseplate with the subsequent overmolding of a rib element on it. We examined the bonding between the additive-manufactured component and the injection-molded component. As bonding strength between the coupled elements is significantly lower than the strength of the material, we proposed five strategies to improve bonding strength. The strategies are optimizing the printing parameters to obtain high surface roughness, creating an infill density in fused filament fabrication (FFF) parts, creating local infill density, creating microstructures, and incorporating fibers into the bonding area. We observed that the two most effective methods to increase bonding strength are the creation of local infill density and the creation of a microstructure at the contact area of FFF-printed and injection-molded elements. This increase was attributed to the porous structures that both methods created. The melt during injection molding flowed into these pores and formed micro-mechanical interlocking.

## 1. Introduction

The modern plastic industry is currently undergoing significant changes, which is dictated by the requirements of Industry 4.0. One of the prominent trends that plastic production must comply with is the trend toward mass individualization, which implies a simultaneous increase in product/process variety while maintaining mass production [1]. The most effective and most established plastic mass production technique is injection molding, which is usually associated with the high equipment cost, mold development that takes a long time, and slow response to possible changes in product design and/or the process itself. At the same time, additive manufacturing (AM), a relatively new plastic processing technique, typically does not involve high costs and is considered a highly versatile process [2,3]. AM can create intricate structures, which are usually hard or even impossible to manufacture with traditional methods. For example, AM can manufacture topologically optimized structures and auxetic structures [4]. AM can also significantly simplify the transfer from the results gained in the lab to the industrial product [5]. However, AM’s bottleneck is the production rate in the long run, which is three to four orders of magnitude slower than that of injection molding [6]. A combination of a relatively high production rate and customized production can be achieved with hybrid technologies, where several plastic processing techniques are combined to manufacture a single part [7,8,9,10]. 

**Figure 1:**
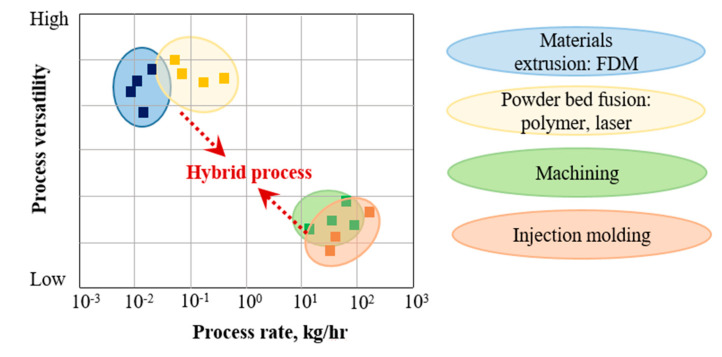
Performance of additive manufacturing (AM), injection molding, and the related hybrid technologies (based on [5]).

The coupling of AM and injection molding can significantly improve product versatility, although at the expense of production rate (Figure 1). However, in cases when an intricate geometry together with high load-bearing capacity are required, traditional manufacturing techniques are not economically feasible. Therefore, Original Equipment Manufacturers (OEMs) use hybrid technologies more and more often to produce polymer and polymer composite parts. For example, TxV Aero Composites recently produced an aircraft storage bin bracket using a hybrid technique that includes overmolding [11]. The areas of application in which a combination of AM and injection molding is economically feasible are most likely to be in medium-scale production. According to [12], the break-even point of using AM (fused filament fabrication, FFF) and injection molding of Acrylonitrile Butadiene Styrene (ABS) is several hundred units, while for selective laser sintering (SLS) of nylon and injection molding, it is approximately 1000 units. This suggests that the number of products for which the combination of AM and injection molding can be economically feasible ranges from several hundred to several thousand units. Based on this, we can assume that the best application areas for the above-mentioned hybrid technology could be medical devices, soft robotics [13,14], sports goods, medium-scale production of automobiles, aerospace, etc. 

The literature analysis proves that the combination of AM and injection molding is mostly recommended for medium-volume production with the high necessity of personalization. Laptoiu et al. [12] proposed combining AM and injection molding to produce customized bi-material hand splints. The splint’s rigid part was 3D-printed from polylactic acid, while the soft part was injection molded from fast-curing silicon. Interestingly, the authors called the second manufacturing step “injection molding”, while in fact, it was most probably reactive injection molding. Kim et al. [15] proposed a combination of automated tape placement of long glass fiber (GF)-reinforced polypropylene (PP) and injection molding of short GF-reinforced PP for the production of an automobile front bumper component. Brecher et al. [16] produced a hybrid thermoplastic composite truss of tape-based carbon fiber (CF) reinforced blank and 3D-printed PA12 rib structure. 

The possibility of combining AM with injection molding generated various manufacturing concepts. For example, a rigid substrate can be produced in the first step by injection molding, and additional structural elements on the surface of the substrate can be produced by AM, or vice versa [8]. Another interesting manufacturing concept is that AM is used to produce 3D preforms that serve as a reinforcement for a composite structure. Since AM builds the product layer by layer, even the most complex 3D preforms can be made. Chou et al. [17] produced three different 3D-orthogonal preforms from short CF-reinforced ABS. These preforms were further infused with a silicon elastomer system. Although the authors demonstrated the feasibility of this concept, they highlighted the necessity to scale up AM production to satisfy mass production needs. In 2017, the Markforged company patented a similar concept, in which a preform is created from continuous CFs; then, this preform is overmolded with a thermoplastic matrix [18]. Another original hybrid manufacturing concept under the name “injection printing” was recently proposed by Kazmer and Colon [19]. The idea of injection printing involves manufacturing a 3D-printed shell and injecting a large volume of polymer into the shell’s cavities. This way, production speed was increased by an average factor of 3.2 relative to conventional 3D printing. One more hybrid manufacturing concept that combines AM with injection molding is so-called “rapid tooling” (RT) for injection molding. RT makes it possible to create an injection mold within a few days or even hours at a reasonable price [20,21], thus significantly reducing the time to market of injection molded parts. This way, RT facilitates faster conformation of the manufacturing systems to changing market demands, rapid reaction to changes in product design, etc.

Although a combination of AM and injection molding is promising for the personalized medium-scale production of polymer and polymer composites, the weak point is bonding between the components, which is usually much weaker than the strength of a single-piece part. A number of papers focus on improving bonding strength by optimizing the processing parameters [22,23,24,25], surface treatment [26], or creating mechanical bonding between the coupled components [11,27,28,29]. However, all the above-mentioned studies focus on a bonding problem within a single manufacturing process and do not cover hybrid technologies. Mechanical bonding is especially effective when chemical bonding is insufficient or when the bond would degrade during operating conditions. Mechanical bonding between two components can be improved in different ways. For example, Laptoiu et al. [11] demonstrated the effectiveness of cylindrical connection points when joining an additively manufactured substrate with an overmolded silicon component. Another option is to create so-called “infill patterns” in the base plate along the contact area. During overmolding, the molten polymer fills the pattern and thus creates a micro-mechanical interlock along the contact line.

The literature review suggests that the modification of FFF processing parameters will influence the tensile properties and the quality of the printed parts. However, fewer papers have investigated the bonding strength between printed and injection-molded parts. Therefore, our goal is to investigate and compare the effectiveness of different mechanical interlocking methods between the additive manufactured and overmolded parts and to prove that bonding strength can be increased. Our study analyzes the bonding mechanism by examining the effect of various FFF printing parameters (layer height, printing speed, printing orientation), infill density, local infill density, microstructures, and the use of carbon fibers (CFs) on bonding strength. We made the specimens from ABS. Our results can help choose the right bonding enhancement method in overmolding FFF printed parts.

## 2. Materials and Methods 

### 2.1. Filament Preparation 

We selected ABS GP35 (Terluran GP35, INEOS Styrolution, Cologne, Germany) as our primary material, since this material can be processed by both injection molding and 3D printing. The ABS pellets were dried in an oven at 80 °C for 4 h. The filaments with a diameter of about 1.75 mm were extruded with a twin-screw extruder (LTE 26-44, LabTech Scientific, Samutprakarn, Thailand). The temperature profile of the extruder was 210, 215, 220, 225, and 230 °C (from the feed section to the die). The screw speed was set to 80 rpm.

### 2.2. Carbon Fiber-Reinforced Pellets

We produced the CF-reinforced ABS pellets by manually mixing ABS GP35 pellets with 15 wt % 6 mm long CF (PX 35, Zoltek, Nyergesújfalu, Hungary). The mix was extruded. The processing temperature was in the range of 185–225 °C, and the screw speed was 80 rpm. The CF/ABS extruded strands were cooled with a cooling fan and pelletized into granules of approximately 3 mm long and then dried. 

### 2.3. Fused Filament Fabrication (FFF) Printing Process

We produced 80 mm x 80 mm x 2 mm preforms (base plates) from the ABS filament. We printed the preforms using an FFF printer (Craftbot plus, CraftUnique Ltd., Budapest, Hungary). The nozzle temperature was 250 °C, and the platform temperature was 105 °C. Four parameters were varied: printing speed (20 mm/s, 40 mm/s, 60 mm/s, and 80 mm/s), layer height (0.1 mm and 0.2 mm), printing orientation (0/90° and 45/−45°), and infill density (50%, 75%, 80%, 90%, 95%, and 100%). All the inputs were saved in the STL file. The end product of the slicing process was done with Craftware software. The 3D file illustrates the layer path and arrangements. 

### 2.4. Surface Roughness Measurement

We measured the surface roughness (Ra) of the preforms with a contact-based surface roughness profilometer (Mitutoyo SJ 400, Mitutoyo Ltd., Kawasaki, Japan). The different printing parameters resulted in different Ra values. We also evaluated the surfaces by studying the microscopic image of the FFF-built parts with a digital microscope (Keyence VHX-5000, Keyence corp., Osaka, Japan). The area of surface roughness (Sa) of printed preforms was also measured with a laser scanning wide-area 3D measurement system (Keyence VR-5000, Keyence corp., Osaka, Japan).

### 2.5. The Overmolding Process

**Figure 2:**
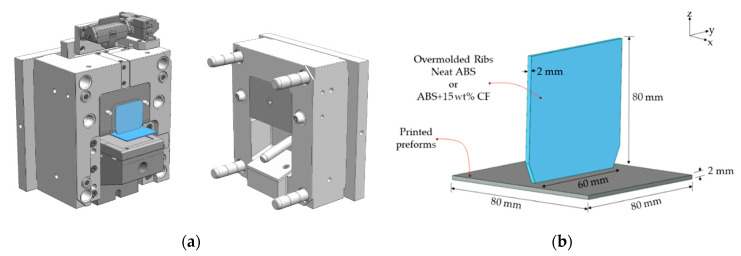
(**a**) Injection mold with the slider for overmolding; (**b**) Overmolded specimen containing a base plate and a rib.

After the printed preforms were cooled, we overmolded two sets of specimens on preforms: one set with pure ABS pellets and another with CF/ABS pellets. The injection molding machine was an Arburg Allrounder 420C 1000-290 (Arburg GmbH, Lossburg, Germany). The melt temperature was set to 240 °C, and the mold temperature was set to 60 °C. We used a newly developed mold [8] with a slider (Figure 2a), which can accommodate the printed preforms and will create a rib in the middle. The shape of the final overmolded part is shown in Figure 2b. It consists of a base plate (former preform) and an overmolded rib. The mold can also be used without the preform to manufacture the reference specimen in which both elements (a base plate and a rib) are injection-molded together.

### 2.6. Tensile Tests

**Figure 3:**
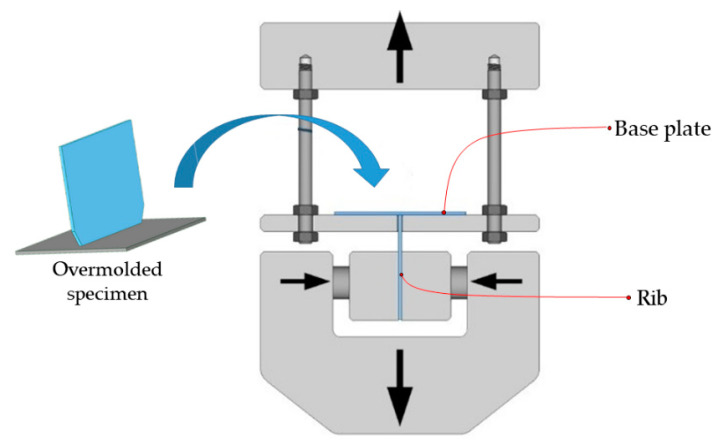
A specimen that consists of a base plate and a rib and a special grip for the measurement of bonding strength.

To find the relationship between the technological parameters of FFF and bonding strength, we carried out tensile tests at room temperature and a relative humidity of 50%. Eight specimens were tested for each set, and a travel speed of 5 mm/min was used for the experiments. We used a grip of our own design (Figure 3) on a universal testing machine (Zwick Z020, ZwickRoell Group, Ulm, Germany). The lower grip of the machine is the standard grip with a maximum load of 20 kN, and the upper head consists of a platform hanging on four screws from the crosshead. The base plate of the sample was laid on the platform with a gap, and the rib was fixed with the clamp. The gap was 1 mm larger than the rib on each side. The nominal connecting surface of the preform and the rib was 120 mm^2^. Its actual value depended on the surface finish and features.

## 3. Experimental Plan

The bonding strength between the printed and overmolded parts can be improved by modifying the surface roughness because “rough” surfaces have a greater area for bonding [16,17,18,19]. Another method is to create protruding ridges or a deeply pitted surface, which will have a comparatively larger contact area. Therefore, the mechanical force for pulling out the overmolded parts from these pits or ridges lead to a substantial increase in the work of debonding [26,27,28,29]. If the overmolded material thoroughly penetrates the pits or completely surround the ridges, it may not be possible to pull out the ribs without fracturing them.

**Figure 4:**
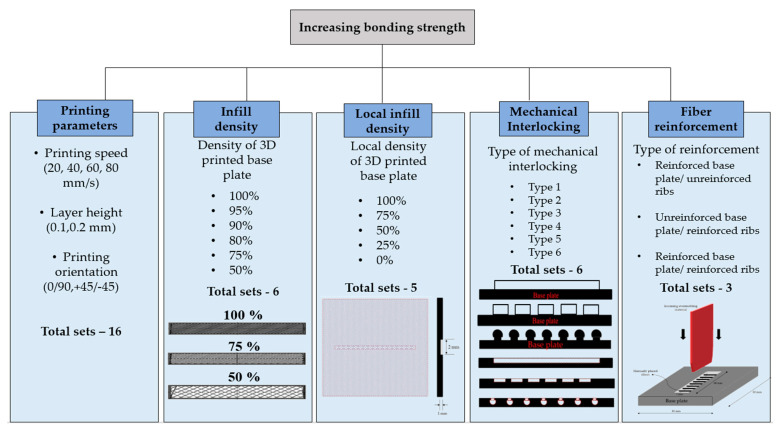
Methods of bonding strength improvement, which involve modifying the base plate.

In this study, we examined five different ways to improve the bonding strength between 3D-printed and overmolded elements. The first method increases bonding strength by increasing the surface roughness of the 3D-printed baseplate—this was achieved by the optimization of FFF processing parameters. We varied printing speed (20, 40, 60, and 80 mm/min), the height of a layer (0.1 and 0.2 mm), and printing orientation (0/90 and +45/−45 deg.). The second method of increasing bonding strength is creating a so-called “infill”, which is practically a grid-like structure. We examined infill densities in the range of 50 to 100%. The third method is creating a “local infill density”, where a triangular pattern of reduced density is made only in the contact area of the base plate and the overmolded rib. The width of the contact area was 2 mm; the examined infill densities ranged from 0 to 100% with a step of 25%. The fourth method we investigated was mechanical interlocking. We addressed six types of mechanical interlocking patterns (Figure 4). The fifth method was introducing fiber reinforcement in the contact area and the ribs. We created three different combinations: reinforced preform/unreinforced ribs, unreinforced preform/reinforced ribs, and reinforced preform/reinforced ribs. The constant and variable parameters for each method are presented in Table 1. 

## 4. Results and Discussion

### 4.1. Effect of Printing Parameters on Bonding Strength

The specimens were divided into 16 sets with different combinations of FFF processing parameters: printing speed (*v*_print_), layer height (*h*_layer_), and printing orientation (*O*_print_), as shown in Table 2. Eight samples per set were printed, and their results were averaged. The hypothesis we want to verify is that surface roughness affects the bonding strength between the overmolded and the printed parts. The FFF processing parameters which were kept constant are platform temperature (105 °C), nozzle temperature (250 °C), nozzle diameter (0.4 mm), and printing pattern (parallel lines). The results (roughness, bonding strength, and build time) are presented in Table 2. The term “build time” means the total time from the start of the print to the nozzle’s return to the initial position. 

The data presented in Table 2 indicate that the surface roughness (Ra) is very high compared to traditional manufacturing processes, such as injection molding. In the plastic injection molding industry, a mean Ra value of 0.05 µm is recommended [30], while our FFF-printed samples had Ra ranging from 2.28 to 21.28 µm. Contact-based surface roughness measurement yielded a very high standard deviation between the Ra values of the same set. This might be because of the difference in the direction the probe was moving (along printing orientation 0° or printing orientation 90°). Therefore, we only used laser-scanned area surface roughness measurement (Sa) for further analysis.

**Figure 5:**
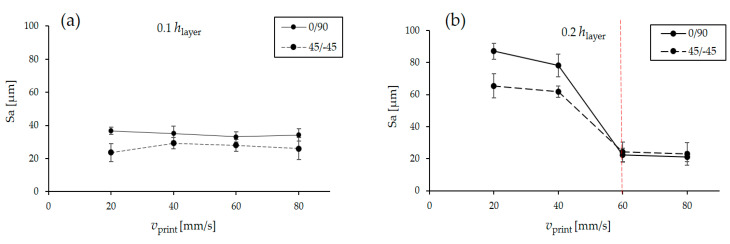
Comparison of surface roughness (Sa) at different printing speeds at 0.1 mm (**a**) and 0.2 mm (**b**) individual layer thickness.

We found that at a layer height of 0.1 mm, printing speed does not affect surface roughness irrespective of printing orientation (Figure 5). This could be because each raster width is very small at 0.1 mm, so the air gap between the raster remains the same in any printing speed. On the other hand, at a layer height of 0.2 mm, it is evident that surface roughness is much higher than in the case of a layer height of 0.1 mm with both printing orientations. However, we observed a sudden decrease in surface roughness above a printing speed of 40 mm/s. This phenomenon can be attributed to the way the layers are deposited at lower speeds. At lower speeds (20 and 40 mm/s), the layers are deposited and cooled very slowly, which leads to more definite patterns, which in turn leads to higher surface roughness. At 0.2 mm, the raster width is greater, and at higher speeds, these rasters merge together, eliminating air gaps. At higher speeds (60 and 80 mm/s), the deposited filaments were stretched and the layers were smoothed; thus, surface roughness was lower. 

We used an ANOVA to evaluate the significance of each factor (processing parameter) to the response (Table 2). In the FFF process, the surface roughness (Sa) is the average of the highest peak and the valley of the surface profile. Table 3 represents the ANOVA results for surface roughness. Printing speed has the most significant effect on surface roughness (44.02%), followed by printing orientation (3.68%) and layer height (3.41%). Thus, higher speed makes the surface smoother.

Meanwhile, bonding strength was also influenced more by printing speed (41.67%), as shown in Table 4**.** The time required to create the preform by FFF is build time. The ANOVA results for build time are shown in Table 5. Printing speed has the highest effect on build time (66.99%), which is followed by layer height (32.92%), and printing orientation does not affect build time. It is obvious that printing speed affects build time. An increase in layer height reduces the layers required to print the part; therefore, it reduces nozzle head movement. It is observed that none of the selected parameters shows the significance of 95%; therefore, more rigorous controls are needed to achieve the desired surface roughness and bonding strength. 

**Figure 6:**
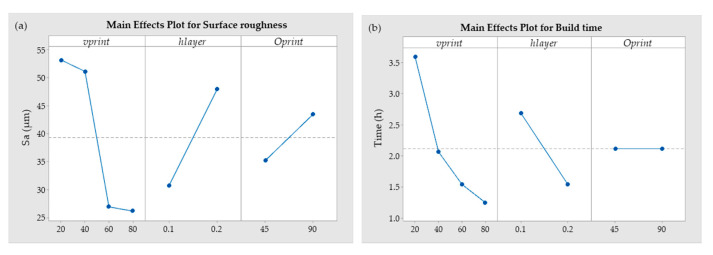

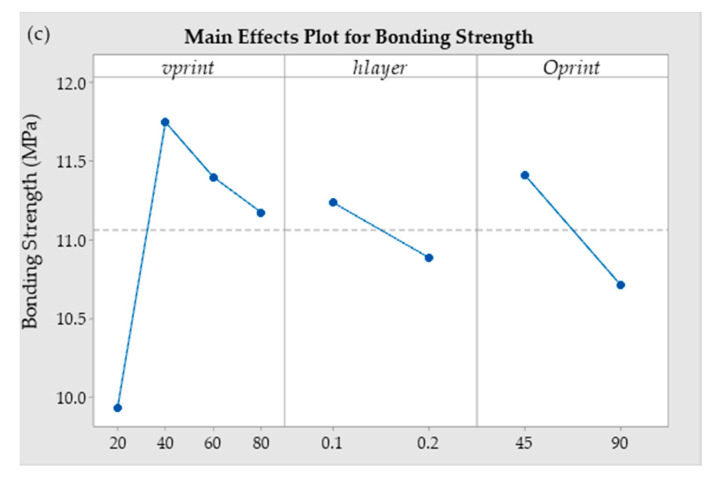
(**a**) Main effects plots for mean surface roughness; (**b**) Main effects plots for mean build time; (**c**) Main effects plots for mean bonding strength.

The main effects plot shows that as the layer thickness increases from 0.1 to 0.2 mm, the surface roughness (Sa) increases as well. Thicker layers form higher “peaks” on the surface, and also the gap between two consecutive layers of the 3D-printed part increases; therefore, the surface roughness increases (Figure 6a). We also observed that the surface roughness decreases drastically when the printing speed exceeds 40 mm/s. This is because at a higher speed (60 and 80 mm/s), the deposited filaments were stretched, and the layers were smoothed. In addition, increasing the layer height reduces the build time. This is not only because more material is deposited but also because at a layer height of 0.2 mm, the gap between the two layers is larger, so printing finishes faster. Printing orientation does not affect build time (Figure 6b). 

This section’s ultimate goal was to find a correlation between surface roughness and bonding strength. However, practically, we observed no evident correlation between these parameters. A possible reason could be the high melt temperature during overmolding. The polymer at 240 °C melts away the base plate’s top layer, irrespective of surface roughness. Although bonding strength plots show a correlation with all parameters, the standard deviation of bonding strength is higher than that of the changes in the mean values. Therefore, the observed bonding strength between a rib and a base plate has negligible correlation with the surface roughness of the base plate. This motivates us to use other strategies and build more complex preforms so that the effect of bonding strength is more evident.

### 4.2. The Effect of Infill Density on Bonding Strength

**Figure 7:**
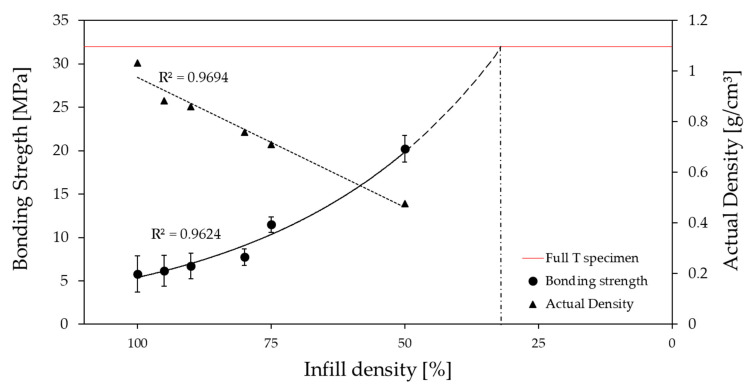
Effect of infill density on bonding strength as an exponential function and corresponding actual density as a linear function. The red line represents the measured experimental bonding strength value for the fully injection molded reference specimen (32 MPa). Symbols are experimental results; black solid and dotted lines are model predictions.

The preforms were printed with various FFF parameters, as shown in Table 1. As expected, the infill density has an evident effect on the bonding strength between the base plate and the overmolded rib. Infill density has an obvious impact on the macrostructure of the 3D-printed preforms. Figure 7 shows bonding strength and density as a function of infill density. The bonding strength of the 50% infill density specimen (20.2 MPa) is approximately three times higher than that of the 100% infill density specimen (5.8 MPa). The amount of material consumed increased with the increase in infill density. Thus, base plates with 50% and 100% infill density are 6.2 g and 13.2 g, respectively, which shows a 53% increase in mass. In addition, increasing the infill density increases the overall printing cost due to the more prolonged printing time (0.78 h for 50% infill and 1.49 h for 100% infill) and the increased amount of material consumed.

**Figure 8:**
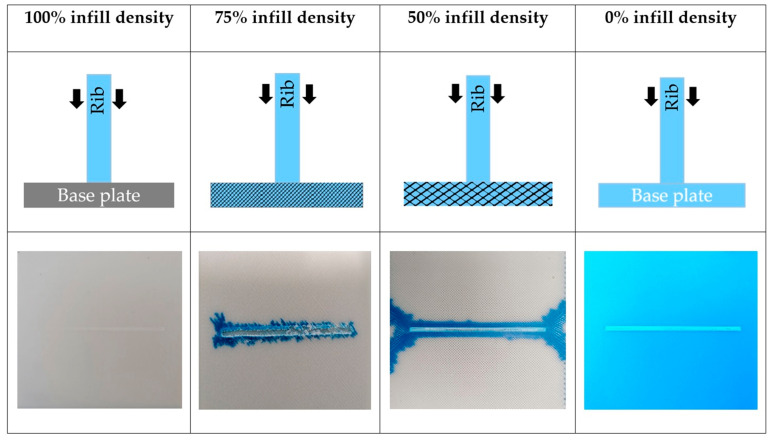
Illustration of 3D-printed preforms with 100%, 75%, 50%, and 0% infill densities, and images of debonded base plates at these infill densities.

At an infill density of 100%, the rib comes into contact with the base plate, and there is simple adhesion between them (Figure 8), which leads to lower bonding strength (5.8 MPa). Lower infill densities cause bigger gaps in the preform or, in other words, an increase in porosity. A comparison of preforms printed with 100%, 75%, and 50% infill densities is shown in Figure 8, and this clearly shows the changes in porosity. At 0% infill density, bonding strength is basically equal to the bonding strength of the fully injection molded specimen (32 MPa). The bonding mechanism between these two limits depends on the porous content of the base plate and the penetration level of the incoming melt. So, with a 50% infill-density base plate (50% porous), a considerable amount of melt flows into these grid-like structures and forms a nearly full injection molded specimen. Therefore, there was an exponential increase in bonding strength (20.2 MPa). Due to the limits of the structural stability of FFF printed parts, infill densities below 50% cannot be created. As the bonding strength increases exponentially, we expect that the highest bonding strength of 32 MPa can theoretically be reached even before 0% infill density (Figure 7). Therefore, at around 30% infill density, these grid-like structures are very weak, which allows the melt to flow through them and melt them easily.

Consequently, the highest bonding strength of 32 MPa could be reached for the base plate with an infill density between 30% and 0%. Practically, it is not achievable as at an infill density below 50%; the base plate breaks when it is removed from the FFF platform.

When the ABS melt (blue color) was overmolded on to the low infill density preforms, the melt penetrated these pores and resulted in better bonding between the base plate and the rib. However, the melt also flows through the pores and comes out through the preform’s edges (Figure 8). Therefore, we let the overmolding melt flow in a certain region; then, we blocked its path and contained it inside the preform. Hence, the local infill density was created and evaluated (explained in Section 4.3). 

### 4.3. Effect of Local Infill Density on Bonding Strength

**Figure 9:**
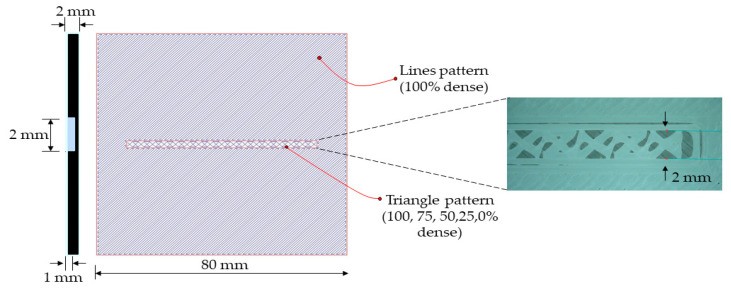
Illustration of local infill densities at the center of the base plate, where the overmolding rib will be in contact.

To stop the melt overflowing through the preform, we altered the infill density only along the ribs’ contact area while keeping the other area of the preform at 100% density (Figure 9). As the commonly used slicing software was not efficient enough to create different infill densities in different areas, we used Slic3r. This way, we were able to create any infill density (100%, 75%, 50%, 25%, and 0%) with triangular local infill patterns in a specific area for the overmolded rib.

**Figure 10:**
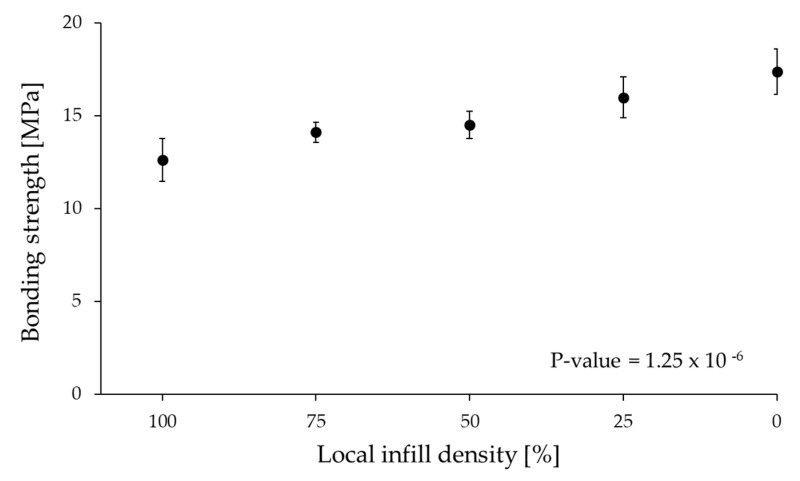
Effect of local infill density on bonding strength and the p-value from the ANOVA test.

The preforms were printed with constant and variable FFF parameters, as mentioned in Table 1. A local infill density of 0% practically means a groove in the base plate. This groove is the bonding area. In all the other cases, grid-like structures with different local infill densities were created in the contact area. These grid-like structures are usually weaker than the full-density preform; that is why we call them a “weak layer”. When the rib is debonded, the weak layer with the local infill area breaks with the rib. Maximum bonding strength was observed at 0% infill density, where the contact surface area with the rib is the highest (429 mm^2^) and the rib has direct contact with the preform. Figure 10 shows that there is a little but gradual increase in bonding strength from 100% (12.6 MPa) to 0% local infill density (17.4 MPa). The disadvantage of the melt overflowing in the preforms was also rectified by containing the overmolding melt inside the boundaries of the created local infill density part. We used the ANOVA test to determine the significance of local infill density on bonding strength. Since the *p*-Value was less than 0.05, local infill density had a statistically significant effect on bonding strength at a confidence level of 95%.

### 4.4. Effect of Microstructure on Bonding Strength

We have proved that bonding strength increases with contact surface area. Thus, another possible solution to improve bonding between 3D-printed and overmolded elements is to create ridge and pit elements in the bonding area of the preform. These microstructures create a larger contact surface between the base plate and the overmolded rib. An increased contact surface area helps the overmolding melt to flow into these undercuts on the base plate to form mechanical interlocking. The mechanical force pulling out the overmolded parts from these ridges or pits lead to a substantial increase in the work of debonding. If the overmolded material thoroughly penetrates the pits or completely surrounds the ridges, it may not be possible to pull out the ribs without fracturing them.

**Figure 11:**
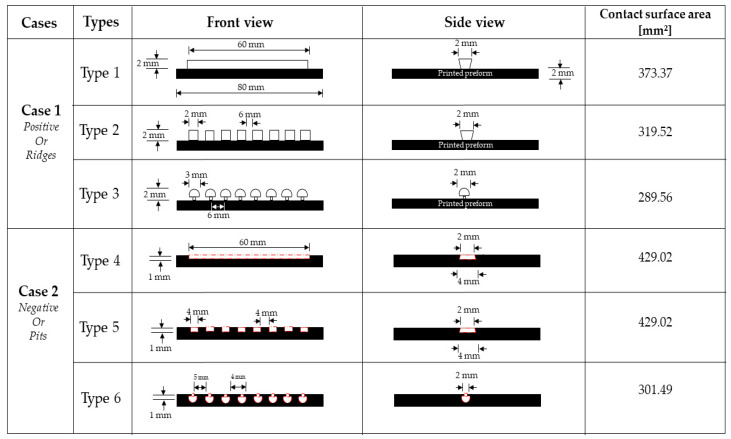
Illustration of the ridges on and the pits in the FFF printed preforms along with their dimensions in mm and their corresponding contact surface area with the overmolded ribs.

We explored several designs we found in the literature to establish a suitable microstructure with an increased contact surface area between the preforms and the ribs [31]. However, we narrowed the possibilities down to few simple geometries due to the limitations of FFF and injection molding. We created geometrical features (positive and negative) on the top layers of the preform, where the overmolded rib is in contact with it (Figure 11). 

**Figure 12:**
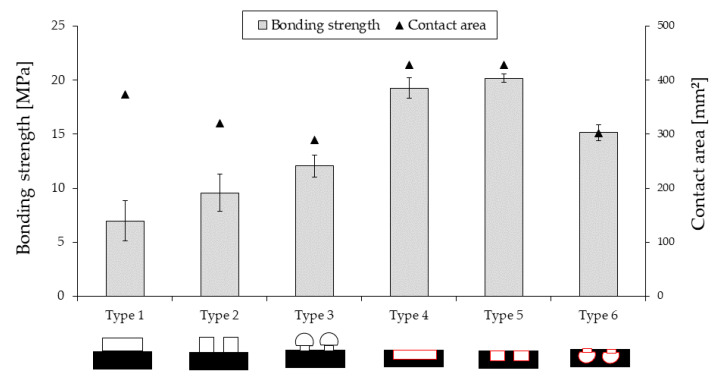
Effect of microstructures on bonding strength. Symbols represent the actual contact area between the base plate and the ribs. The bars represent the experimental results for bonding strength.

We considered two simple types of microstructures: ridges (positive) and pits (negative), and each one has three different patterns. The shape of the microstructures and the corresponding contact area of these elements significantly affected bonding strength, as the experimental results show (Figure 12). In the first case (ridges), when we analyzed the broken (debonded) samples, we found that the breakage happens between the microstructure and the preform rather than the preform and the ribs. We assumed that the microstructure was debonded easily from the preform because of the weak interlayer adhesion between the printed layers. We compared the results of the positive structures with the negative ones.

In the second case (pits), the overmolding melt thoroughly penetrates the pits with the highest contact area (429.02 mm^2^), and it forms mechanical interlocking. This way, there is no way of pulling out the ribs without breaking the preforms. Here, the contact area has a more pronounced effect than the bonding strength of the printed interlayer. As seen in Figure 12, the highest contact area is observed in Type 4 and Type 5 (429.02 mm^2^) microstructures, and the corresponding bonding strength for these types is the highest, 19.3 MPa and 20.2 MPa, respectively.

### 4.5. Effect of Fiber Reinforcement on Bonding Strength

**Figure 13:**
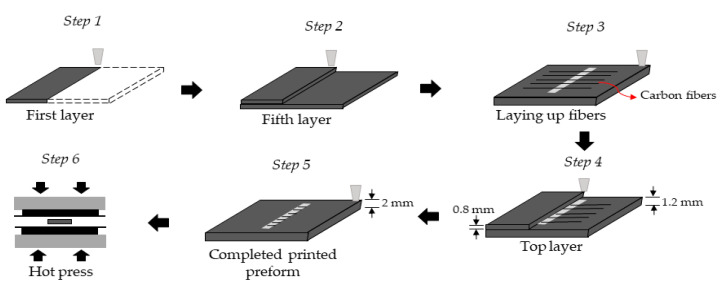
Illustration of the production of the preform with the manually placed carbon fibers.

**Figure 14:**
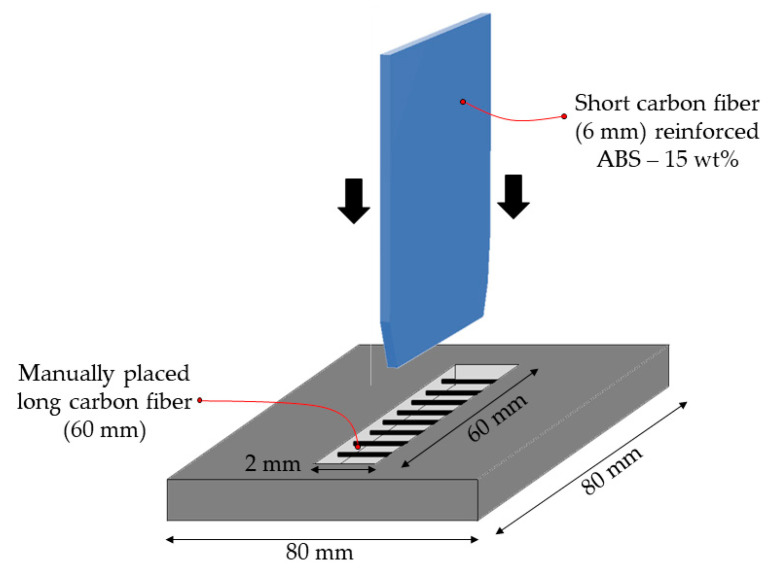
Illustration of overmolding with fiber-reinforced printed preforms.

The concept of introducing fibers in the preforms and/or ribs is the next bonding strength improvement technique. The idea behind it is that the preform can be reinforced locally in the bonding area in the following way: a groove is created in the bonding area while the preform is printed, and then long CFs (60 mm length, 100 µm diameter) are placed across this groove. The bottom of the groove was at a height of 0.4 mm. Then, when the preform was 1.2 mm thick (and consequently, the groove was 0.8 mm deep), eleven fibers were placed manually across the groove, (Step 3—the white area is the groove). After this, the FFF continued to print the remaining 0.8 mm height on top of the CFs on both sides of the groove, as shown in Figure 13**.** After the printing process, the preforms were hot pressed. The final preform can be seen in Figure 14. We anticipated that a kind of mechanical interlocking is formed caused by fiber entanglement.

The prepared short fiber reinforced ABS pellets were used to overmold these locally fiber-reinforced preforms (Figure 14). Four combinations were studied: Unreinforced preforms/unreinforced ribs (ABS/ABS), Unreinforced preforms/CF reinforced ribs (ABS/CF-ABS), Locally reinforced CF preforms/unreinforced ribs (L-CF ABS/ABS), and Locally reinforced CF preforms/CF reinforced ribs. (L-CF ABS/CF-ABS)

**Figure 15:**
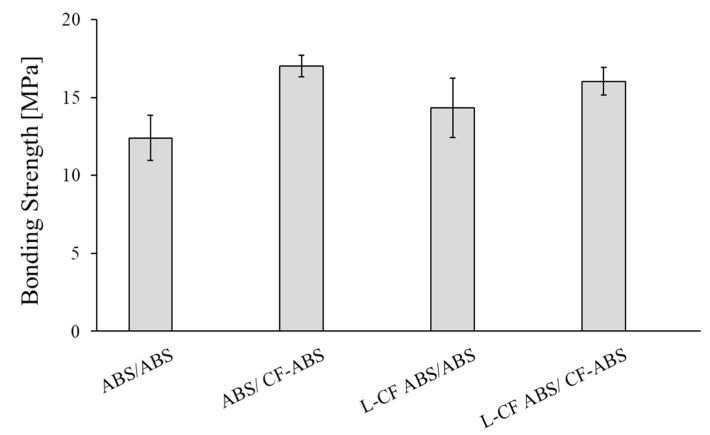
Effect of fiber reinforcement on bonding strength.

As we reinforced the preforms locally with carbon fibers, we anticipated that the incoming overmolding melt should flow under the manually placed CFs in the preform. We expected a mechanical interlocking due to the fiber entanglement of the long fibers in the preform with the ribs’ short fibers. However, the manual placement of CFs in the preform results in poor tension of the fibers. Therefore, these fibers were forced to bend by the overmolding melt, and so, less melt flowed under them. Therefore, the bonding strength between the reinforced preforms and the ribs were not as high as expected (Figure 15).

**Figure 16:**
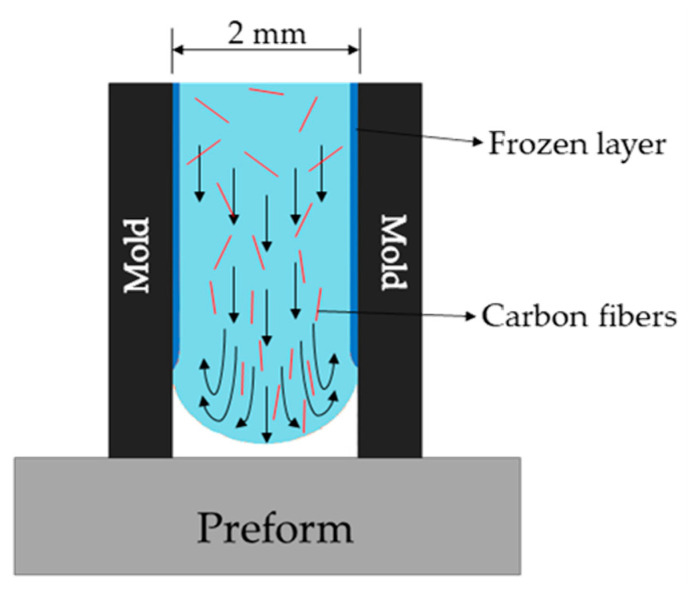
Illustration of fountain flow in injection molding, where the CFs of high thermal conductivity are oriented along the melt flow and concentrated near the base plate.

Nevertheless, the bonding strength between preforms and reinforced ribs were higher than between preforms and unreinforced ribs. This is because of the higher thermal conductivity of CFs. When CFs/ABS melt fills the cavity of the mold, it forms a fountain flow, where the fibers are oriented along the direction of the melt (Figure 16). Consequently, the highly oriented CFs transfer more heat from the melt to the preform. 

### 4.6. Comparison of the Obtained Results

**Figure 17:**
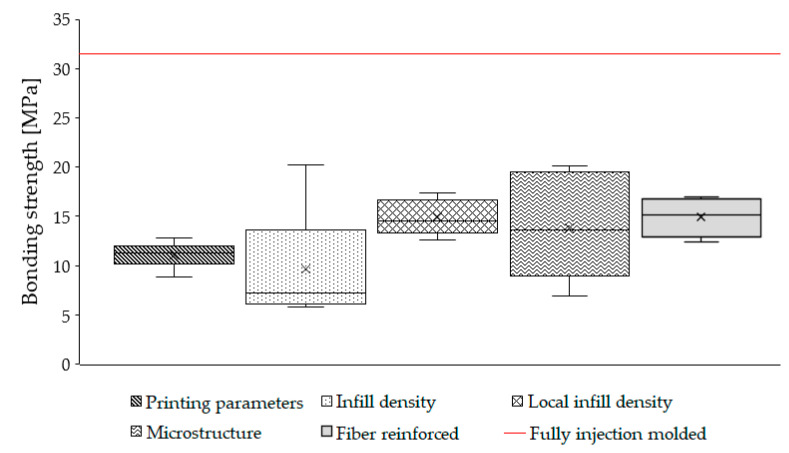
Comparison of the bonding strength of all methods. The red line represents the experimental bonding strength result of the fully injection-molded reference specimen (32 MPa).

In conclusion, except for the varying of printing parameters, all the methods discussed in this article have a significant impact on bonding strength. The bonding strength of all the overmolding specimens is shown in Figure 17 compared to the reference specimen’s bonding strength. The analysis of the obtained results showed that none of the proposed methods of bonding improvement produced the reference bonding strength, which is, practically, material strength. However, the proposed methods facilitate the increase in bonding strength between the printed and overmolded parts significantly. The trend is clear that the modified preforms showed better bonding strength than the unmodified ones. The best results were delivered when the “infill density” or “microstructure modification” methods were used. In these two cases, bonding strength can be increased up to 20.2 MPa, which is an 83% increment compared to the original (plain) preforms (≈11 MPa), and it is much closer to the strength of the reference specimen (≈32 MPa). We obtained adequate results with the “local infill density” and “local fiber reinforcement” methods, which showed 57% and 45% bonding strength increment compared to the original preforms (17.3 and 16.03 MPa, respectively). Varying the printing processing parameters resulted in negligible bonding strength increase. 

### 4.7. Recommendations

Based on the obtained results, we recommend creating microstructures to increase the contact surface area. Moreover, by creating more complex structures with undercuts, mechanical interlocking is possible, which results in higher bonding strength. To rectify the poor interlayer adhesion between the printed layers, we recommend using selective laser sintering (SLS) as an alternative approach, where interlayer adhesion is much stronger than in the case of FFF [31]. The different ways of curing in SLS can contribute to the interlayer cross-linking reaction, which can substantially strengthen interlayer adhesion between adjacent layers by creating covalent bonds across the interface [31]. With AM, the versatility of hybrid technology can be used for mass customization.

Another promising technique to improve bonding strength was to introduce fibers. We placed these fibers manually on the preform. However, we assume that a more sophisticated way of incorporating these fibers could lead to improved bonding strength. One such method is automatically laying up the fibers during the printing stage. The dual extruder FFF printer could pave the way to printing any fibers into the layers where the overmolded melt will contact.

## 5. Conclusions

In this research, we offered a solution for the individualization of high-volume production. We experimented with 3D-printing a baseplate and then overmolding a rib element on it. The bonding strength between the 3D-printed and the injection-molded part plays a key role in determining the strength of the final part. We have suggested five methods to improve bonding strength between the printed preforms and the overmolded ribs.

*Method 1:* The printing parameters (printing speed, layer height, and orientation) significantly affect surface roughness. The highest surface roughness was observed when the printing speed was 20 mm/s, layer height was 0.2 mm, and orientation was 45/−45 degrees). However, surface roughness has little to no impact on bonding strength because the overmolding melt (240 °C) will remove the top layer of the printed preform, irrespective of surface roughness.

*Method 2:* The bonding strength of the 50% infill density specimen (20.2 MPa) is approximately three times higher than that of the 100% infill density specimen (5.8 MPa). In addition, reducing infill density reduces overall printing costs by decreasing printing time and the amount of material consumed. The downside of lowering infill density is that the overmolding melt fills the pores and starts to overflow from the preforms at lower infill densities (e.g., 50%).

*Method 3:* Maximum bonding strength was observed at 0% local infill density, where the contact surface area with ribs is the highest (429.02 mm^2^) and ribs and preform have direct contact without the presence of the grid-like structure.

*Method 4:* As the surface contact area affects bonding strength, it can be increased by creating ridges and pits on top of the preform. 

*Method 4a:* In the case of ridges, the ribs were easily broken from the preform because there was weak interlayer strength between the ridges and the preform. Therefore, debonding contributes more to weak interlayer strength than the larger contact area. 

*Method 4b:* In the case of pits, the melt can easily flow into them and have better mechanical interlocking. In addition, a larger contact surface area means higher bonding strength.

*Method 5:* The incorporation of CFs into the preforms and/or ribs had a minor effect on bonding strength because the expected fiber entanglement did not occur. However, the bonding strength of the reinforced rib specimen is greater than that of the unreinforced rib specimen. This is because more heat from the melt is transferred to the preform through the highly oriented thermally conductive carbon fibers.

In the next stage of research, we will continue to develop Method 4 and optimize microstructures using SLS. We will also investigate the quality of interlayer adhesion to correlate it with the bonding strength of SLS/injection-molded hybrids.

## Figures and Tables

**Table 1:** Constant and Varied fused filament fabrication (FFF) parameters for all examined methods.

Method	Varied Parameters	Constant Parameters
Printing parameters	Printing speed (20, 40, 60, 80 mm/s) Layer height (0.1, 0.2 mm) Printing orientation (0/90, 45/−45)	Infill density—100% Infill pattern—Rectilinear lines Nozzle temperature—250 °C Platform temperature—105 °C
Infill density	Infill density (100%, 95%, 90%, 80%, 75%, 50%)	Infill pattern—Rectilinear lines Nozzle temperature—250 °C Platform temperature—105 °C Infill printing speed—40 mm/s Layer height—0.2 mm Printing orientation—45/−45
Local infill density	Local infill density (100%, 75%, 50%, 25%, 0%) Local infill pattern—Triangular grid Local infill printing speed—10 mm/s	Infill density—100% Infill pattern—Rectilinear lines Nozzle temperature—250 °C Platform temperature—105 °C Infill printing speed—40 mm/s Layer height—0.2 mm Printing orientation—45/−45
Mechanical Interlocking	Microstructure type (6 types) Microstructure printing speed—10 mm/s	
Fiber reinforcement	Reinforcement type (3 types)	

**Table 2:** Design of experiments for different printing parameters and the results.

	FFF Processing Parameters			Results						
Set	*v*_print_ (mm/s)	*h*_layer_ (mm)	*O*_print_ (deg)	Ra (µm)		Sa (µm)		Bonding Strength (MPa)		Build Time (hours)
				Mean	SD	Mean	SD	Mean	SD	
1	20	0.1	0/90	3.35	1.70	36.3	2.16	10.5	1.8	4.71
2	20	0.1	45/−45	7.11	1.79	23.6	5.47	10.2	1.4	4.71
3	40	0.1	0/90	7.66	3.09	35.1	4.54	10.4	1.1	2.64
4	40	0.1	45/−45	9.90	1.92	29.3	3.36	12.9	1.3	2.64
5	60	0.1	0/90	7.96	4.77	33.1	3.09	12.2	1.8	1.91
6	60	0.1	45/−45	9.47	1.25	27.9	3.45	11.7	1.3	1.91
7	80	0.1	0/90	11.06	4.37	34.3	3.73	9.9	1.2	1.50
8	80	0.1	45/−45	11.15	1.44	26.0	6.66	12.1	0.9	1.50
9	20	0.2	0/90	2.28	1.24	87.1	4.93	10.1	1.5	2.49
10	20	0.2	45/−45	21.24	4.02	65.5	7.47	8.9	1.6	2.49
11	40	0.2	0/90	2.38	1.04	78.1	7.12	11.3	0.7	1.49
12	40	0.2	45/−45	20.97	3.85	61.8	3.43	12.4	1.5	1.49
13	60	0.2	0/90	21.28	7.60	22.4	4.13	10.1	1.2	1.17
14	60	0.2	45/−45	15.22	5.18	24.2	6.29	11.6	2.0	1.17
15	80	0.2	0/90	18.61	4.76	21.2	3.04	11.2	3.0	0.99
16	80	0.2	45/−45	20.97	3.44	23.1	7.12	11.5	0.9	0.99

**Table 3:** The ANOVA for surface roughness (Sa) (DF is the Degree of Freedom, Adj. SS–Adjusted Sums of Squares, Adj. MS–Adjusted Mean Squares, *f*-value-is the test statistic used to determine whether the term is associated with the response, *p*-value–is a probability that measures the evidence against the null hypothesis).

Source	DF	Adj SS	Adj MS	*f*-Value	*p*-Value	Contribution
PS	3	0.000935	0.000312	3.00	0.082	44.02%
LH	1	0.000072	0.000072	0.70	0.423	3.41%
PO	1	0.000078	0.000078	0.75	0.406	3.68%
Error	10	0.001039	0.000104			48.89%
Total	15	0.002125				100%

**Table 4:** The ANOVA for bonding strength.

Source	DF	Adj SS	Adj MS	*f*-Value	*p*-Value	Contribution
PS	3	3603.4	1201.1	3.22	0.070	41.67%
LH	1	240.9	240.9	0.65	0.440	2.79%
PO	1	1074.5	1074.5	2.88	0.120	12.43%
Error	10	3729.2	372.9			43.12%
Total	15	8648.0				100%

**Table 5:** The ANOVA for build time.

Source	DF	Adj SS	Adj MS	*f*-Value	*p*-Value	Contribution
PS	3	0.211559	0.070520	2594.56	0.000	66.99%
LH	1	0.103980	0.103980	3825.62	0.000	32.92%
PO	1	0.000000	0.000000	0.00	1.000	0.00%
Error	10	0.000272	0.000027			0.09%
Total	15	0.315810				100%

## Data Availability

Data sharing is not applicable to this article.
